# Practices of and Perspectives on Palliative Sedation Among Palliative Care Physicians in Ontario, Canada: A Mixed-Methods Study

**DOI:** 10.1089/pmr.2023.0081

**Published:** 2024-02-13

**Authors:** Amy Nolen, Debbie Selby, Fahad Qureshi, Anneliese Mills

**Affiliations:** ^1^ Department of Family and Community Medicine and University of Toronto, Toronto, Ontario, Canada.; ^2^ Division of Palliative Care and Sunnybrook Health Sciences Centre, Toronto, Ontario, Canada.; ^3^ Department of Medicine, University of Toronto, Toronto, Ontario, Canada.; ^4^ Department of Family and Community Medicine, Sunnybrook Health Sciences Centre, Toronto, Ontario, Canada.

**Keywords:** end of life, interviews, palliative care, palliative sedation, sedation, symptom management

## Abstract

**Background:**

Palliative sedation (PS) is a therapeutic intervention employed to manage severe and refractory symptoms in terminally ill patients at end of life. Inconsistencies in PS practice guidelines coupled with clinician ambiguity have resulted in confusion about how PS is best integrated into practice. Understanding the perspectives, experiences, and practices relating to this modality will provide insight into its clinical application and challenges within the palliative care landscape.

**Objective:**

The aim is to explore the perspectives of palliative care physicians administering PS, including how practitioners define PS, factors influencing decision making about the use of PS, and possible reasons for changes in practice patterns over time.

**Methods:**

A survey (*n* = 37) and semistructured interviews (*n* = 23) were conducted with palliative care physicians throughout Ontario. Codes were determined collaboratively and applied line-by-line by two independent investigators. Survey responses were analyzed alongside interview transcripts and noted to be concordant. Themes were generated through reflexive thematic analysis.

**Results:**

Five key themes were identified: (1) lack of standardization, (2) differing definitions, (3) logistical challenges, (4) perceived “back-up” to Medical Assistance in Dying, and (5) tool of the most responsible physician.

**Conclusion:**

There was significant variability in how participants defined PS and in frequency of use of PS. Physicians described greater ease implementing PS when practicing in palliative care units, with significant barriers faced by individuals providing home-based palliative care or working as consultants on inpatient units. Educational efforts are required about the intent and practice of PS, particularly among inpatient interprofessional teams.

## Introduction

Palliative sedation (PS) is the practice of administering pharmacological sedation to manage refractory suffering near the end of life.^1^ PS was first described in the literature in the 1990s,^2^ and the terminology and clinical practices related to PS have evolved over time such that there exists significant variability between countries and care settings.^3^

In response, dozens of clinical practice guidelines have been developed by medical associations, palliative care experts, and health care institutions around the world to provide harmonized best practice standards and ethical guidance to clinicians.^4,5^ Regional heterogeneity in PS practices in Canada prompted the Canadian Society for Palliative Care Physicians to produce a consensus-based framework for PS in 2012, and this remains the foremost national guidance document related to PS even 11 years on.^6^

The practice of PS in Canada remains variable in many respects, including PS terminology, medication protocols, indications and frequency of use, and approach to ethical considerations such as PS for refractory existential distress. One study from Calgary, Alberta published in 2019 showed that PS was utilized in 4.2% of all eligible deaths; within this population, 22.2% of patients in the palliative care unit (PCU) received PS, versus only 3.3% of patients dying on hospital inpatient units.^7^

In comparison, a study in Toronto, Ontario showed that 14.6% of patients who died in a PCU during a one-year period received PS, compared with 5% of deaths in a related hospital inpatient unit.^8^ There are also data demonstrating that rates of PS are changing over time in Canada and abroad, which may be due in part to the evolving end-of-life care landscape after the legalization of Medical Assistance in Dying (MAiD) in different jurisdictions.^8–10^

This study aimed to better understand the perspectives of palliative care providers administering PS, including how practitioners define PS, factors influencing decision making about the use of PS, and possible reasons for changes in practice patterns over time, with specific attention to MAiD. This is an explanatory mixed-methods sequential design study that combines data from an online survey and individual interviews with palliative care providers throughout Ontario, Canada.

## Methods

### Participants

Participants were physicians practicing palliative care in any clinical setting in Ontario, Canada. Subjects were eligible to participate if they had been practicing independently for a minimum of five years and were sufficiently proficient in English to complete the survey questionnaire and/or the interview. This study was approved by the institutional Research Ethics Board (REB) at Sunnybrook Health Sciences Centre.

### Data collection

#### Survey

The Ontario Medical Association (OMA) is a professional organization that represents Ontario's physicians; membership is voluntary and members are able to join constituency subgroups relevant to their medical specialty. The OMA Section on Palliative Medicine maintains a mailing list of all members and disseminates regular e-mails to subscribers.

Recruitment for the survey occurred through the Section's March 2022 e-mail newsletter, which was delivered to 1145 members and included a link to participate alongside a description of the study. The survey was administered on the Qualtrics software platform and consent was implied if participants voluntarily proceeded after reading the initial preamble on informed consent. See Supplementary Appendix SA for survey questions.

#### Semistructured interviews

At the conclusion of the survey, respondents were invited to provide their e-mail if interested in participating in the interview component of the study. Participants were also able to self-refer to schedule an interview if they learned of the study through word of mouth. Informed written consent was obtained before the interview and participants were asked permission to audio-record. Interviews took place between March and July of 2022; they were individual semistructured interviews of ∼30 minutes duration that were conducted through the Zoom virtual communication platform.

The interview guide was developed based on review of the literature and experience of the research team, and the guide was amended after the first five interviews. The interviewer was a resident physician with prior experience in qualitative interviewing. Questions focused on how PS was defined within participants' personal practice, frequency of PS, clinical indications for use, and further elaboration as to possible reasons accounting for changes in PS practices. Interviews also explored participants' views on how the 2016 legalization of MAiD in Canada may have influenced their PS practices; results pertaining to this topic are published elsewhere (see Supplementary Appendix SB for final interview guide).^11^

### Data analysis

Descriptive statistics were used to characterize and analyze the survey data. Quantitative survey data analysis occurred concurrently with qualitative data collection, which allowed for an iterative approach.^12–14^ The research team employed “codebook thematic analysis”^15,16^ or “template analysis”^17^ by collaboratively generating a series of codes and subcodes and executing these line-by-line. Each transcript was coded separately by two team members (A.M. and F.Q.); codes were finalized after the first four interviews, at which point saturation was noted.^18^ Reflexive thematic analysis was carried out to generate themes and saturation in this regard was achieved after 12 interviews.^16^

At this point, further recruitment was halted although already-scheduled interviews were completed. The research team felt that there was significant overlap between the survey and interview data thus survey comments were analyzed alongside interview transcripts. This article presents themes related specifically to PS practices; themes addressing the relationship between MAiD and PS were presented in an earlier publication.^11^

## Results

The survey was available between March and May of 2022 through the Section on Palliative Medicine's e-mail newsletter to 1145 members. It was completed by 37 respondents, although not all respondents answered all questions. Demographics of the survey respondents are summarized in Table 1. Ninety percent of respondents reported that they administered PS to 20% or fewer of their patients for the past year.

**Table 1.** Demographics of Survey Cohort

	Survey respondents *N* = 37
Age groups in years, *n* (%) (*N* = 37)	
<30	0
30–44	21 (57)
45–60	10 (27)
>60	6 (16)
Sex, *n* (%) (*N* = 37)	
M	17 (16)
F	20 (54)
Years working in palliative care, *n* (%) (*N* = 35)	
5–10	15 (43)
11–20	9 (26)
21–30	10 (29)
>30	1 (3)
Location of practice (multiple options per respondent), *n*	
Academic site	26
Community site	15
Other	3
Types of palliative care service (multiple options per respondent), *n*	
Home based	17
Outpatient clinic	18
Inpatient consults	21
PCU/hospice	23
Part of acute care	1
Percentage of patients for whom the practitioner initiated PS for the past 12 months, *n* (%)	
0%	1 (3)
1–10%	24 (73)
11–20%	6 (18)
21–30%	2 (6)
>30%	0 (0)
Indications for use of PS (multiple options per respondent), *n*	
Refractory delirium	33
Refractory dyspnea	26
Refractory pain	16
Existential distress in the absence of delirium	15
Other^a^	6
Most common indication for using PS, *n* (%)	
(31 respondents only)	
Refractory delirium/agitation	26 (84)
Refractory dyspnea	4 (13)
Existential distress	1 (3)

Not all participants responded to all questions.

^a^ Other = hemorrhage, refractory seizure, refractory vomiting

PS, palliative sedation.

Respondents identified refractory delirium/agitation as the primary indication for PS (34.4%), with refractory dyspnea (27.1%), refractory pain (16.7%), and existential distress (15.6%) also common indications. Less common indications included refractory nausea, seizures, and catastrophic hemorrhage. Quotations from survey responses are attributed to Respondents (R) 1–37.

Semistructured interviews were conducted with 23 palliative care providers. One provider was deemed to be ineligible based on length of time practicing palliative care and was subsequently excluded from analysis. For the demographics of the interview participants, please see Table 2. There was partial overlap between survey and interview participants (14 survey participants also completed interviews); the interviews allowed the researchers to gain greater contextual understanding of current practices, and provided increased depth and richness of data.

**Table 2.** Demographics of Interview Cohort

	Interview participants *N* = 22
Age in years, median (range)	45 (34–69)
Sex, *n*	
M	10
F	12
Years working in palliative care, median (range)	12.5 (5–39)
Location of practice, *n*	
Home based	9
Outpatient clinic	7
Inpatient consult	12
Palliative care unit/hospice	9

When physicians were asked to comment on the use of PS both within their personal practices and their wider communities of practice, five main themes emerged. Quotations from interviews are attributed to Participants (P) 1–22. For all the themes presented hereunder, please see additional supporting quotations under Table 3.

**Table 3.** Themes and Associated Quotations (Interview Participant = P, Survey Respondent = R)

Theme	Subtopic	Quotation(s)
Theme 1: Lack of standardization in practices	Variability in frequency of use of PS	P5: “I would say maybe out of every hundred people, maybe three, three to five, like as little as that.” P14: “Not even once a month, maybe once every two to three months, maybe as frequent as that. But sometimes it, it could be once every four to six months.”
	Changes frequency of use due to increased awareness of palliative care as a discipline	P1: “[…] palliative sedation as a concept has evolved as palliative care has, in that people have gotten more comfortable with talking about end of life and dying in general, then that opens conversations around some of the more complex aspects of symptom management.” P2: “I think people are more aware and they talk amongst each other […] I mean, I'm thinking back to a lecture I saw like 15 years ago [on PS] where everyone was like, ‘Really? I'm not going do that!’”
	Lack of standardized eligibility criteria and practice protocols	P3: “[As a resident] there was zero standardization, a lot of it was dependent on the religious background, the values of the particular physicians. […] I found that very challenging to navigate.” R36: “Standardization in practice is needed. There is huge variation in use and protocols/doses.”
Theme 2: Differing definitions	PS as a secondary effect of symptom control	P2: “I'm maybe not reporting it accurately because I'm not counting the people that I've thrown on a midazolam pump in the last 24, 48 hours of life because they're so distressed.” P23: “We start with medications that are for delirium. And if symptoms persist, we move to more sedating medications, but I don't think it gets labeled as palliative sedation. So we're probably doing it a lot for delirium at end of life.”
	PS defined by intent and outcome	P16: “Palliative sedation…is a form of ongoing sedation used to control intractable and intolerable symptoms, for a patient who is end of life in the last maybe hours or even days usually. And that involves lowering their level of consciousness, usually, almost always, in order to manage their symptoms. And [palliative sedation] is different from managing symptoms where I use other medications, mindful of trying to minimize sedation and other side effects.” P20: “I use it more broadly whenever I'm using anything where I know that part of my intention is to sedate to try to manage some of their symptoms. […] When I use sedating antipsychotics to manage terminal delirium, I do consider that a form of palliative sedation… if you truly were to ask me, what is my intention with these medications? Is it to treat the delirium or is it to sedate them so that they seem to be less agitated? If I'm being honest, I think a big component of it is the sedation piece.”
	Discomfort with label “palliative sedation”	P4: “It happens all the time in acute care that people are given very sedating medications to manage delirium. It's essentially palliative sedation, but I don't think anybody labels it that. It's probably happening more [in a PCU], just cause we're recognizing it and labeling it as that.” P22: “Perhaps people are doing palliative sedation without labeling it as palliative sedation, in the acute care setting, particularly if it's a non-palliative care provider, you know, they may be treating patients, sedating patients, but not necessarily using that term.”
Theme 3: Logistical challenges	Lack of familiarity with PS among interdisciplinary team	P6: “[…] there's a lack of understanding among physicians, nursing staff, allied health, everybody about, you know, the principle of double effect and, what is and is not appropriate medical therapy. So there's a large knowledge gap that is definitely a barrier, and a fear about giving the last dose of whatever medication, you know, a sedative, opioid, whatever. They're afraid of, whether it's consequences from their direct supervisor or from their college or whoever. But definitely there's fear about that kind of thing.” P15: “Over the years I think sometimes a barrier—and something I guess I learned from a long time ago—is to ensure that all team members are on side…it's important to recognize that not all team members may see palliative sedation in the same way. And some people have sort of conscientious objections to palliative sedation, believing that they may be hastening death and, can experience distress at some of the findings…now I've always ensured that all team members are comfortable with the concept of palliative sedation and are willing to go through with it.” P16: “There's a very memorable case…on our ICU … I set it up so that we would be able to, you know, deliver palliative sedation and make her comfortable. And the nurse who was looking after her, we wrote all the orders, went away, gave it a couple of hours to get everything in place and came back and the nurse hadn't done anything, had not followed any of the orders. And just essentially kind of told us that she, well, first of all, when we were talking about it, she stood there and said, well, this is killing a person. And, you know, she sort of offered her objection. I had offered to talk to her about the difference between this and MAiD, and offered to even find another nurse for her. And she just kind of wouldn't interact. And I left with the assumption that this was all going be taken care of, only to come back to her passive resistance, which was to have done nothing…And it's very interesting because it occurred, you know, after the time MAiD was present and it was very definitely connected to that person's perception that what we were really doing was ‘under the cover, back door’ MAiD and wasn't prepared to listen to that as an, an entirely different system, you know, or entirely different treatment modality.”
	Challenges unique to community practice	P5: “I find it very difficult to do palliative sedation at home because to increase the meds in that rapid fashion and to expect families to be orchestrating that is really out of the question.” P7: “The main barrier is availability of medications in the community. If I want to start palliative sedation, it often will take, you know, 8 to 12 hours to get all the medications in place.”
THEME 4: Perceived “back-up” to MAiD		P9: “I think some […] view palliative sedation as a form of MAiD, like if they haven't qualified for MAiD because of capacity. So then the family or the POA wants PS. I find that very challenging cause I do not view them as the same thing.” P17: “[the] delineation between the two is becoming less and less clear and a lot of patients will say things like, oh, well it's okay if I can't get MAiD, then I'll just use palliative sedation as a backup kind of thing. And it's like, well, […] that's not the indication for palliative sedation. But they'll sometimes pressure us or sort of advocate for the use of it, even if it's in the situation where we might otherwise not have even offered it. And that becomes a difficult conversation.”
THEME 5: Tool of the MRP		P6: “I think probably the palliative care physicians are the ones who are comfortable talking about it. And so, and probably have a little bit more license to do so when they're most responsible physician, as opposed to one of many consultants. Um, so probably why it would be a little bit higher in a PCU rather than in a hospital that, you know, palliative care doctors can't be MRP. […] Um, so yeah, I think that probably has something to do with it when palliative care doctors are in control, it's probably more likely to happen than when there's a different MRP involved.” P7: “And so having the discussion that sedation may also be given for existential distress might also increase the rates. And those conversations are more likely to be had in a PCU where someone is sort of talking to someone they know who's the MRP versus in acute care.” P11: “And because, the team is the MRP […] I would say that that's a reason we might have seen a threefold increase in the context that the increased awareness we've talked about, you know, over the last several years.” P14: “My experience is that as a PCU staff versus an inpatient consult staff, it's a different approach because you're no longer the MRP as an inpatient consultant […] as you may know, to even get a palliative care consult […] there's often lag times and huge barriers.”

ICU, intensive care unit; MAiD, Medical Assistance in Dying; MRP, most responsible physician; PCU, palliative care unit; POA, power of attorney.

### THEME 1: Lack of standardization

All participants were asked around the typical frequency of PS in their practice. There was marked variability between participants, irrespective of practice setting. One survey respondent even reported never using PS in the past 12 months.

*When I'm on-call on the weekend for palliative care, I regularly start palliative sedation on at least 2 or 3 or 4 patients on a weekend….it is common…I would say that for people that are in hospital that I look after when I'm on-call, that are dying in hospital from their disease and not from MAiD, I would say that palliative sedation is probably involved in 50%.* [P3]

*I've actually only seen [PS] done once in 20 years… Maybe twice.* [P21]

A subset of practitioners commented on utilizing PS relatively often and speculated as to why the practice might be increasing in frequency. Participants perceived greater understanding and acceptance by the general public of palliative care as a discipline, and thus an increase in willingness to discuss end-of-life care options including PS.

*I think people are probably requesting palliative sedation more than they used to be, but I think it is part and parcel of a greater understanding and knowledge of the role of palliative care, such that it's like patients and caregivers are demanding it of their health workers.* [P11]

Aside from overall frequency, practitioners also detailed significant variability with respect to PS provision and practices more generally. A lack of standardized eligibility criteria and practice protocols resulted in inconsistencies in how and when PS was initiated, which was a source of frustration for some physicians.

*I've really struggled with why we don't have a Canadian standard for palliative sedation criteria…[Physicians] have just kind of done things over the years in certain ways, without a certain standard. And it is just physician preference for when palliative sedation is initiated….it is creating discordant practice across the country.* [P11]

One participant was able to share that Ontario specifically has comparatively little standardization. This physician—and one other—acknowledged the limits of a standardized approach, but highlighted that it might be particularly useful for clinicians with less experience in providing PS.

*When I was working in Quebec what I found was that Palliative sedation became much more rigorized […] There were forms we had to fill […] 5,6 to 10 pages to fill out […] You had to get consent: “I'm doing Palliative Sedation because of this symptom, these are the things we've already tried, these are the medications we are using for Palliative Sedation.” When did we start sedation? When did the patient stop eating? When did we stop IV fluids? When did the patient stop drinking? When did the patient actually die? What was the reaction of the team? The family members? Were there family meetings about all of this?” […] I think the document and the whole systematic need to do that was a step in trying to standardize things. Of course we know it's never standard. You have to tweak this and tweak that depending on the patient's response. So my perspective is that it made things easier for people who are not as knowledgeable or comfortable with palliative sedation, because there was almost a guide there.* [P14]

*I think the way it was presented before, like the whole guideline thing, I think it scared people off initially. When it was “terminal sedation” and there were all these guidelines.* [P2]

### THEME 2: Differing definitions

Participants expressed further variability with respect to continuum of clinical practices encompassed by the term “palliative sedation.” There was a range of opinions voiced regarding scenarios where patients are sedated as a by-product of symptom control efforts. Several participants expressed uncertainty regarding such scenarios and whether they might—in the moment or in retrospect—constitute PS:

*It's one of those things where we often don't realize we're doing palliative sedation until we've done it. You're just giving someone, you know, nozinan or midazolam to calm their agitation. Then all of a sudden you're on a pathway towards deep, continuous palliative sedation*. [P7]

A minority of clinicians discussed that such scenarios, while ethically or phenomenologically blurred, would not constitute PS unless it had been formally discussed in a declarative manner:

*I think this comes back to the definition of palliative sedation therapy, you know, we have the example where we're using sedatives to manage a delirium, not for the intent of palliative sedation, but as a side effect, you're going to sedate somebody with an antipsychotic for example. And so there's a fine line. There is a real grey zone between: are we actually formally doing a palliative sedation process? Or are we just continuing with our care and turning up the Continuous Ambulatory Delivery Device (CADD) pump, for example, because the person is much more delirious and essentially we're sedating them because that's what the side effect is? And* technically speaking, that's not palliative sedation because you haven't had the conversation*, but in essence, you're essentially sedating a patient with a treatment, purposely sedating them. So there's a real grey zone there*. [P19] (emphasis added)

*Many people will be sedated by the time we've achieved a certain level of comfort, but* I haven't directly put them on a palliative sedation pathway. [P5] (emphasis added)

Even when physicians had differing opinions on specific clinical scenarios—for example, the use of atypical antipsychotics for the treatment of delirium, which was the most frequently discussed—there was a shared sentiment that PS was fundamentally defined by the intention of the clinician:

*[Palliative sedation] is not giving someone medications for the treatment of agitation…that's just appropriate treatment of agitation. It is not someone happening to become drowsy after receiving an analgesic. It is where you measure your success by the patient being asleep.* [P6]

Finally, several participants highlighted that, irrespective of definitions or intent, physicians may be hesitant to label their practice as “palliative sedation.”

*Sometimes people won't call it palliative sedation, because palliative sedation is a term that is loaded…they'll call it symptom management or they'll call it something else. If you use midazolam at 0.5 mg/hour, that's not palliative sedation dosing, but 2 mg/hour is? Which is not correct. What is correct is intent…it's not about dose, it's about intent. […] You could ask 10 palliative care doctors about palliative sedation and some of them would say they never do it. And that may be true, but it also may be true that they call it something else.* [P3]

### THEME 3: Logistical challenges

Many participants spoke about discomfort among the interdisciplinary team members as a universal barrier to PS that crossed all practice settings. There was a sense that a lack of familiarity with the practice fuelled fear in staff and reluctance to participate in PS. Specifically, many practitioners noted that team members were reluctant to use PS due to lack of familiarity and concerns about hastening death.

*Barriers will be the familiarity or lack thereof among my interdisciplinary team members with palliative sedation on what it is, and quite honestly, actually, how it differs from MAiD…that's actually become a barrier to its use because there's clarity around conscientious objection for MAiD, but when my colleagues aren't really differentiating or seeing the differences with palliative sedation then there tends to be hesitation around its use. And so it's almost trickier to use palliative sedation sometimes than MAiD.* [P1]

*Many people, both laypersons and allied health workers continue to confuse palliative sedation with MAiD, making it a challenge to propose and get buy-in from the team for palliative sedation at times.* R29

Difficulty with the comfort of the interprofessional team was particularly pronounced for physicians providing consultative palliative care for inpatients in acute care or intensive care. In extreme instances, this was noted to result in obstructive behavior.

*I would say that barriers in my inpatient practice to doing palliative sedation would be someone who's not on our PCU…it's through the palliative care consult team, and we're seeing someone on a ward that's not our home turf, so to speak, you know, you're working with an interprofessional team where palliative care is not the basis of the work on that ward.* [P11]

*It's familiarity for the nurses. I think nurses in acute care are used to pulse, blood pressure, respiration, temperature,* etc. *Sort of that kind of monitoring as opposed to how deeply is the person asleep. You know, I think if a physician is not trained in palliative care, they haven't got that level of comfort with sedation as well, I would suggest.* [P5]

In the community setting, participants struggled with the availability of resources to initiate PS. These included human resources (such as adequate nursing for routine monitoring and rapid up-titration of sedation), and difficulty obtaining medications and medical supplies in a timely manner.

*I would say that the second barrier is timely delivery of, in my case, in the home setting, timely delivery of the medications. So we work with [the community pharmacies], and they've been very good about instituting more recently, a four-hour turnaround to get supplies in the home, if it's something more urgent. So that's been helpful, but with COVID and the limitations on nursing staff, that's been another limitation more recently. We've had to, ourselves as physicians, sometimes start the CADD pumps ourselves, which is not common, but it becomes a barrier just in terms of timeliness of care.* [P19]

### THEME 4: Perceived “back-up” to MAiD

Several participants felt PS was at times viewed by patients and families—or even other health care providers—as a “loophole” if a patient was not deemed eligible through the formal MAiD process.

*I noticed more families requesting it when a patient has not qualified for MAiD.* R23

*[…] On our team, I feel like this was more of an issue when MAID was first legalized, but we felt at times there was this misconception by referring services that if there was a whiff of, ‘they want MAiD, they can't consent so come do palliative sedation’. […] or this perception that palliative sedation was ‘MAiD light’, and we wanted education on that, that there's criteria for initiating palliative sedation.* [P23]

As evident earlier, the desire by families and other clinicians for PS to be enacted when MAiD was not—or was no longer—an option, raises unique procedural and ethical issues.

### THEME 5: Tool of the most responsible physician

All participants were asked to comment on the Toronto study^8^ as a part of their interview—and were explicitly asked to speculate as to why rates of PS in acute care remained stable, whereas rates in the PCU at the same hospital increased. Although some participants highlighted logistical factors, many stressed that performing PS was either easier or more appropriate if executed by a palliative care physician acting as the most responsible physician (MRP).

*I would also not be comfortable, to be honest with you, doing a consult on a patient palliatively in the hospital and, and looking at palliative sedation and then not transferring to the PCU—unless it was so severe that I'd have to do it instantaneously and there was no bed in the PCU.* [P5]

*And when we are the MRP, we sort of feel a bit more personally empowered to have those conversations or to make those decisions to do palliative sedation, as a group, right? […] sometimes the goals are not so clear depending on who the MRP is.* [P10]

These sentiments likely compound with the obstacles discussed in Theme 3 wherein interprofessional team members based in acute care may be both unfamiliar with, and hesitant to participate, in PS. However, these participants emphasized the uniqueness of the MRP role in deciding to proceed with palliative sedation.

## Discussion

In this mixed-methods study, we sought to understand the evolving perspectives of palliative care practitioners utilizing PS, including how they define the practice, barriers or challenges, and factors that influence frequency of use. Practitioners focused on the lack of uniformity in PS practices, as well as significant variability in how PS is defined. Challenges included confusion and hesitancy among interdisciplinary team members, misconceptions about how PS might be employed in situations where MAiD was not an option, and timely access to medications and equipment in the community. Participants also detailed reluctance to either execute PS or label clinical practices as PS in settings where palliative care physicians were involved as consultants, rather than the MRP.

An important finding is the degree of variability in how PS is defined by medical professionals, thus contributing to the divergence in its practice and reporting across health care settings. There is a wide breadth of literature examining the language used to described PS, but a single consensus is lacking. Variations in terminology include intermittent (or respite) sedation versus continuous sedation,^19^ or proportionate/“gradual” sedation versus continuous deep sedation for “rapid” induction of unconsciousness.^20^

In a Cochrane review, PS is described as sedation achieved by sedating drugs not designed to treat the underlying condition or symptom (primary PS), or by drugs that have some effect on the underlying symptom and with a secondary effect of causing sedation (secondary PS).^21^ Participants in this study described difficulty distinguishing in practice between “primary” and “secondary” PS, which at times generated confusion over whether PS had been employed.

There was disagreement over the use of medications with sedating side effects, particularly for the management of delirium: a minority of participants were adamant that this was not PS; many voiced ambiguities (perhaps articulating “secondary” PS); and one specifically included such management in their definition of PS, reasoning that sedation was at least part of their intention behind using these medications. Such inconsistencies point to confusion among palliative care providers about best practices regarding how and when to utilize PS.

The prevalence of PS varies widely across care settings and geographical regions—including as a result of social, political, and cultural factors.^5,22,23^ A population-based study in Belgium reported an overall prevalence of continuous deep sedation of 13% in 2013.^24^ A similar study in Switzerland showed that 24.5% of all patients with non-sudden deaths received PS, with a higher prevalence for patients dying in PCUs.^25^ Rates of continuous deep sedation at PCUs in East Asian countries have been reported as 10% in Japan, 16% in Korean, and 22% in Taiwan,^26^ whereas Brazilian acute care settings have an estimated prevalence of 54.2%.^27^

In Calgary, Canada, a 2019 study found that PS accounted for 3.3% of deaths in acute care, 4% of deaths in hospice, and 22.2% of deaths in PCU.^7^ In Toronto, Canada, one study at a tertiary care hospital reported that 5.2% of patients in acute care and 4.7% of patients in a PCU received PS for a 12-month period in 2015/16; after legalization of MAID in mid-2016, these numbers changed to 14.6% (in PCU) and 5% (in acute care) at the same institution.^8^

This heterogeneity in practice prevalence was evident among the participants in the study at hand, with many utilizing PS relatively rarely and a small number initiating PS more frequently (with one participant estimating the use of PS in up to 50% of patients at end of life). The respondents expressing greater comfort with PS tended to practice in the inpatient setting, most often a PCU, and this is consistent with literature documenting a higher prevalence of PS in PCU settings.

Possible reasons can be found in this study: hospital settings in general have better resources (i.e., medications, delivery systems, and personnel), thus eliminating some of the barriers associated with home-based PS; PCUs are typically staffed by specialist palliative care physicians acting as MRP; and interdisciplinary staff are in turn more comfortable with PS. Conversely, staff in acute care tend to be less familiar with the practice and may resist PS in an effort to prolong life through active medical management, even if PS aligns with the patient's goals of care. Staff may also misunderstand the intent of PS and resist due to concerns about shortening survival or being used as “back-up” for patients unable to receive MAiD.

Several respondents commented on a lack of standardized practice protocols in Ontario. Internationally, numerous guidance documents have been created for the past two decades in an attempt to harmonize practice and serve as guidance to regional health bodies and institutions attempting to generate policies.^28–32^ Within Canada, there are both national and local guidance documents for PS produced by regional palliative care bodies and individual institutions.^6,33–36^

Local guidelines can result in improvements to clinical practice; they have also been argued to promote uniformity in clinical practice^37^ and reduce fear of medicolegal repercussions.^22^ Nevertheless, there are inconsistencies with respect to recommendations about timing of initiation of PS, medication protocols, and approach to artificial nutrition and hydration. The wide variability with respect to PS definitions and practices described earlier may speak equally to the need to develop protocols and the inherent difficulty in doing so.

More generally, the results of this study demonstrate the need for education efforts pertaining to PS, whether in the form of practice guidelines or other educational interventions. For the past two decades, the College of Family Physicians of Canada and the Royal College and Physicians and Surgeons have developed palliative medicine subspecialty training programs in parallel. More recent graduates are, therefore, likely to have exposure to advanced palliative medicine practices such as PS during their training. In addition, several participants discussed increased personal comfort with PS as a result of growing professional and public awareness.

Thus, education efforts may be particularly important for physicians without specialist palliative care training, and in nonpalliative care settings, as confusion and concern on the part of interprofessional teams was described a significant barrier to performing PS in these settings. Clarifying the process and intent of PS, dispelling stigma, and introducing standardized practice tools^38^ are important to ensuring high-quality palliative care for Ontarians, particularly in light of the evolving array of end-of-life care interventions.

## Limitations

This study only reports on data from Ontario and there may be further interprovincial variability. Similarly, data regarding practice location within Ontario was not collected, so it is not possible to comment on any geographical variability or trends within the province. Finally, even though the available results were concordant with interview data, the survey component only had *n* = 37, which is a small response rate compared with the number of physicians who theoretically had access to the newsletter.

## Conclusion

This study explored palliative care providers' experiences with PS, particularly for the five years after the legalization of MAiD. It presented the following themes: (1) lack of standardization, (2) differing definitions, (3) logistical challenges, (4) perceived “back-up” to MAiD, and (5) tool of the MRP. There was significant variability in terms of how participants defined PS and its frequency of use, which is consistent with available literature internationally.

Physicians expressed greater comfort and ease with PS when practicing in PCUs, with significant barriers described by participants providing home-based palliative care or working as consultants on in-patient units. These results demonstrate the need for greater educational resources and interventions, particularly among inpatient interprofessional teams, where there was often confusion surrounding the nature and goals of PS.

## Supplementary Material

Supplemental data

Supplemental data

## Abbreviations Used

CADD: Continuous Ambulatory Delivery Device
MAiD: Medical Assistance in Dying
MRP: most responsible physician
OMA: Ontario Medical Association
PCU: palliative care unit
PS: palliative sedation
