# Supplementary material for: Practices of and Perspectives on Palliative Sedation Among Palliative Care Physicians in Ontario, Canada: A Mixed-Methods Study
Source: Palliat Med Rep. 2024 Feb 13;5(1):94–103. doi: 10.1089/pmr.2023.0081 (PMC10898238; doi:10.1089/pmr.2023.0081)
Supplement: Supplemental data [file Supp_AppSA.pdf]

Appendix A: Survey Questions

Question 1. What is your age?
- · < 30 years old
- · 30-44 years old
- · 45-60 years old
- · > 60 years old
- · Prefer not to say

Questions 2. What is your gender?
- · Male
- · Female
- · Non-binary / third gender
- · Prefer not to say

Question 3. How many years have you been practicing palliative care?
- · 5-10 years
- · 11-20 years
- · 21-30 years
- · > 30 years

Question 4. What proportion of your medical practice is provision of palliative care?
- · < 25%
- · 26-50%
- · 51-75%
- · >75%

Question 5. Where is your practice located? Choose all that apply.
- · Academic centre
- · Community site
- · Rural centre
- · Other (please specify)

Question 6. What type of palliative care service do you provide? Choose all that apply.
- · Home-care
- · Outpatient / Ambulatory clinic
- · Inpatient consults
- · Palliative Care Unit / Hospice
- · Other (please specify)

Question 7. In the past three months, approximately how many deaths have there been among the patients for whom you personally provided palliative care?
- · < 10 deaths
- · 10-25 deaths
- · > 25 deaths

Question 8.
For the purposes of this study, we define palliative sedation according to the Canadian Society Palliative Care Physicians' Framework for continuous palliative sedation therapy in Canada, specifically:
1) The use of (a) pharmacological agent(s) to reduce consciousness
2) Reserved for treatment of intolerable and refractory symptoms
3) Only considered in a patient who has been diagnosed with an advanced progressive illness
4) Usually considered only in patients in whom death is expected within two weeks or less.

Thinking back over the past year, for what percentage of your patients did you administer palliative sedation at the end of life?

- None
- 1-10%
- 11-20%
- 21-30%

Question 9. What are the reasons you consider using palliative sedation for your patients?

- Refractory delirium / agitation
- Refractory shortness of breath
- Refractory pain
- Existential distress in the absence of delirium
- Other(s) (please list)

Question 10. What is the most common reason you use palliative sedation? (free text):

Question 11. Do you think the frequency of your use of palliative sedation has changed in the past 5-6 years?

- Significantly less frequent
- Slightly less frequent
- Same frequency
- Slightly more frequent
- Significantly more frequent

Question 12. If applicable, what reasons do you think have contributed to this change?

Part 2. Some studies in both Canada and Europe have found a change in the use of palliative sedation after legalisation of assisted dying. The final section of the survey will explore this trend.

Question 13. Have you had patients who have accessed Medical Assistance in Dying (irrespective of whether you had a specific role in the MAID process or not)?

- Yes
- No

Question 14. Have you participated in MAID as either a primary provider or a secondary assessor?

- Yes
- No

Question 15. Within your community, have you noticed a change in the frequency of use of palliative sedation since the legalization of MAID?

- Yes
- No
    - Question 15.i. If yes, has the frequency of use of palliative sedation increased or decreased?
    - Question 15.ii. What do you think is contributing to this change? (we are interested in as many ideas as you wish to share)

Question 16. Within your personal practice, is your use of palliative sedation different since the legalization of MAID?

- Yes
- No
    - Question 16.i. If yes, has your frequency of use of palliative sedation increased or decreased?
    - Question 16.ii. What do you think is contributing to this change? (we are interested in as many ideas as you wish to share)

Question 17. Do you have any additional thoughts to share about Palliative Sedation that we have not asked about?
