# Supplementary material for: Practices of and Perspectives on Palliative Sedation Among Palliative Care Physicians in Ontario, Canada: A Mixed-Methods Study
Source: Palliat Med Rep. 2024 Feb 13;5(1):94–103. doi: 10.1089/pmr.2023.0081 (PMC10898238; doi:10.1089/pmr.2023.0081)
Supplement: Supplemental data [file Supp_AppSB.pdf]

Appendix B: Semi-Structured Interview Guide:

1. Demographic Information

- Can you tell us your age and gender?
- How long have you been providing palliative care for?
- What kind of palliative care practice do you have? (PCU/hospice, inpatient/acute, outpatient, home)

2. We want to know how palliative sedation features into your practice, generally speaking. Can you tell me how you define palliative sedation and in what circumstances you might consider it?

- What kind of symptoms most commonly lead to palliative sedation?
- Do you have any sense of how frequently you perform palliative sedation?

3. Have you encountered any barriers in performing palliative sedation?

4. Have you had any experience with MAiD - either direct or indirect?

- Have any patients you've been caring for requested or received MAiD?
- Have you acted as an assessor or a provider? If not, how do you typically help patients access MAID if they request it?
- If discussing MAiD with a patient, do you discuss palliative sedation? Why or why not? Do you find other practitioners do/don't do this?

5. Thinking back to your practice over five years ago -- do you think you provided palliative sedation more or less frequently as compared to now?

- What do you think might account for this change?
- Do you think any of these changes relate to the implementation of MAiD (eg. greater discussion of EOL choices, social/family reasons)

6. Thinking about palliative care in general, do you think that rates of PS would increase or decrease following MAiD legalisation?

- Why do you think this?

7. I'm going to summarize the results of a recent study conducted at a major Canadian hospital. The researchers found that, following the implementation of MAiD, the rate of palliative sedation at their palliative care unit increased significantly, from about 5% of all deaths to almost 15% of all deaths. However, in acute care at the same site, the rate remained stable at ~5% both pre and post legalization. Based on your experience, what do you think might have contributed to these findings?

- Thinking first of the rise in the PCU, what do you think may be contributory factors?
- Now thinking of the stability of the PS rate in acute care, what do you think can explain this?

8. Do you have any other thoughts or ideas related to these topics?
